# Anodal tDCS over the primary motor cortex improves motor imagery benefits on postural control: A pilot study

**DOI:** 10.1038/s41598-017-00509-w

**Published:** 2017-03-28

**Authors:** Elodie Saruco, Franck Di Rienzo, Susana Nunez-Nagy, Miguel A. Rubio-Gonzalez, Philip L. Jackson, Christian Collet, Arnaud Saimpont, Aymeric Guillot

**Affiliations:** 1 Univ Lyon, Université Claude Bernard Lyon 1, Laboratoire Interuniversitaire de Biologie de la Motricité (EA 7424, LIBM), F-69622 Villeurbanne, France; 2 Universidad de Alcalá, Unidad de Fisioterapia. Campus Universitario, Ctra. Madrid Barcelona, 28801 Alcalá de Henares, Madrid Spain; 3 Facultad de Ciencias, UNED, Departamento Automática e Informática, Paseo Senda del Rey 9, 28040 Madrid Spain; 4 Université Laval, École de Psychologie, Ville de Québec, Québec Canada; 5 Centre Interdisciplinaire de Recherche en Réadaptation et Intégration Sociale, Québec, Canada; 6 Institut Universitaire de France, Paris, France

## Abstract

Performing everyday actions requires fine postural control, which is a major focus of functional rehabilitation programs. Among the various range of training methods likely to improve balance and postural stability, motor imagery practice (MIP) yielded promising results. Transcranial direct current stimulation (tDCS) applied over the primary motor cortex was also found to potentiate the benefits of MIP on upper-limb motor tasks. Yet, combining both techniques has not been tested for tasks requiring fine postural control. To determine the impact of MIP and the additional effects of tDCS, 14 participants performed a postural control task before and after two experimental (MIP + anodal or sham tDCS over the primary motor cortex) and one control (control task + sham tDCS) conditions, in a double blind randomized study. Data revealed a significant decrease of the time required to perform the postural task. Greater performance gains were recorded when MIP was paired with anodal tDCS and when the task involved the most complex postural adjustments. Altogether, findings highlight short-term effects of MIP on postural control and suggest that combining MIP with tDCS might also be effective in rehabilitation programs for regaining postural skills in easily fatigable persons and neurologic populations.

## Introduction

Posture is the ability to maintain an adequate body alignment relative to the gravitational vector^1^. From a functional viewpoint, postural control includes balance, which refers to the dynamics of the body preventing falls^1^. The possibility of completing every-day tasks is inherent to this basic function of human motor control. A lack of postural control leads to a loss of autonomy and a reduction of daily activities affecting the quality of life^2^. Therefore, in the case of severe motor impairments, recovering postural functionalities is one of the main focuses of rehabilitation programs^3^.

Among the various range of training methods used to improve balance and postural stability, motor imagery as well as action observation provided promising results^4^. Motor imagery is the internal representation of a movement without engaging its actual execution. The benefits of motor imagery practice (the repeated use of motor imagery, MIP) on performance have been widely reported in sport and rehabilitation domains^5,6^. In the specific context of postural control, Choi *et al*.^7^ provided evidence that MIP enhanced motor performance during a weight-shifting task. They revealed that MIP significantly decreased the time required to perform the postural task, although to a lesser extent than physical training. In a recent study, Taube *et al*.^4^ showed that MIP might contribute to both promote learning of postural tasks and improve dynamic balance in unstable and non-predictable environments. MIP has further been found to activate the neural regions involved in gait planing^8^, and might therefore contribute to enhance postural control in easily fatigable and physically impaired populations such as elderly persons^9,10^ and stroke patients^11–14^. Specifically, decreased anteroposterior and mediolateral sway, as well as increased holding balance durations on one leg, were measured after MIP during static postural tasks (maintaining a stable stance). Benefits from this method were also recorded for dynamic components of postural control (i.e., remaining stable while performing movements, such as during locomotor tasks). MIP effects on motor performance are frequently interpreted as an account of the neurofunctional equivalence with the physical practice of the same movement^15^. A handful of neuroimaging studies demonstrated that these types of practice shared overlapping brain activations, including the primary motor cortex [M_1_]^16–20^. MIP may thus affect activity-dependent plasticity (i.e., the capacity of neurons to adjust their connectivity to the cognitive/behavioral demand), the neural correlate of the motor learning process^21^. Motor imagery has also been shown to increase the motor evoked potentials and decrease the depolarization threshold of cortical motor regions^22–24^, thus matching the neural excitability changes elicited by physical practice, and certainly promoting motor learning.

Facilitation of motor learning through modulations of the cortical excitability can also result from transcranial direct current stimulation (tDCS), which is a noninvasive brain stimulation method^25^. Neural excitability increases induced by anodal tDCS potentiates inter-neurons communication during physical practice (i.e., increased synaptic gain), thus facilitating motor learning^26^. This might explain the positive impact of anodal tDCS applied during physical practice on sequential/visuomotor tasks, reaction times and functional performances^27^. Interestingly, applying anodal tDCS over M_1_ during motor imagery of a hand movement was found to increase Mu desynchronization, a neural signature of movement execution, imagination and observation^28^. Anodal tDCS might thus facilitate activity-dependent neuroplasticity within the neural networks recruited during motor imagery, thereby accounting for MIP effects on performance. Foerster *et al*.^29^ confirmed the enhancement of motor performance through MIP paired with anodal tDCS applied over M_1_. While MIP alone tended to improve handwriting, additional gains were recorded when MIP was combined with anodal tDCS. Saimpont *et al*.^30^ finally reported greater performance increase after MIP combined with anodal tDCS compared to both MIP and tDCS alone in a sequential task.

Spurred by the results mentioned above, the present study was designed to investigate whether anodal tDCS applied over M_1_ might contribute to potentiate the benefits of MIP on a postural task. As the effects of tDCS alone on postural skills have been the focus of previous studies^31,32^, we hypothesized that MIP alone and MIP paired with anodal tDCS might contribute to improve performance on a weight-shifting task.

## Results

### Motor imagery ability data

Participants’ KVIQ mean visual score (±SD) was 3.46 (±0.70), while their KVIQ mean kinesthetic score was 3.42 (±0.72). Mean total KVIQ score was 3.44 (±0.61).

The comparison of vividness scores on the 5-points Likert scale (Wilcoxon's paired tests) after MI training under MI_tDCS_ (3.28 ± 0.72) and MI_sham_ (2.92 ± 0.82) was not statistically significant (V = 18, p = 0.11).

### Motor performance data

**Figure 1:**
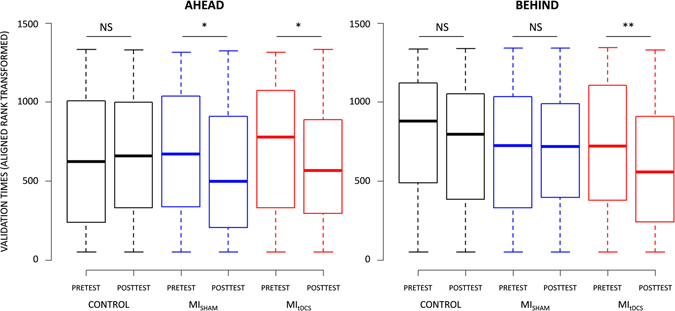
Effect of Control, MI_SHAM_ and MI_tDCS_ conditions on Ahead and Behind targets validation.

The analysis of validation times revealed a CONDITION × TEST × PLAN interaction (F_(2,1235)_ = 7.51, p < 0.001, partial η^2^ = 0.03). As shown in Fig. 1A, a decrease from the Pretest to the Posttest of validation times of Ahead targets was recorded under both MI_tDCS_ (Pretest: 4.17 s ± 1.19, Posttest: 3.98 s ± 1.39; p_(ART)_ = 0.02) and MI_sham_ (Pretest: 4.18 s ± 1.27, Posttest: 3.97 s ± 1.36; p_(ART)_ = 0.03), but not for the Control condition (Pretest: 4.16 s ± 1.58, Posttest: 4.13 s ± 1.47; p_(ART)_ > 0.05). Furthermore, we observed a decrease in validation times from the Pretest (4.37 s ± 1.99) to the Posttest (3.93 s ± 1.34) for Behind targets under MI_tDCS_ only (p_(ART)_ = 0.01, Fig. 1B).

**Figure 2:**
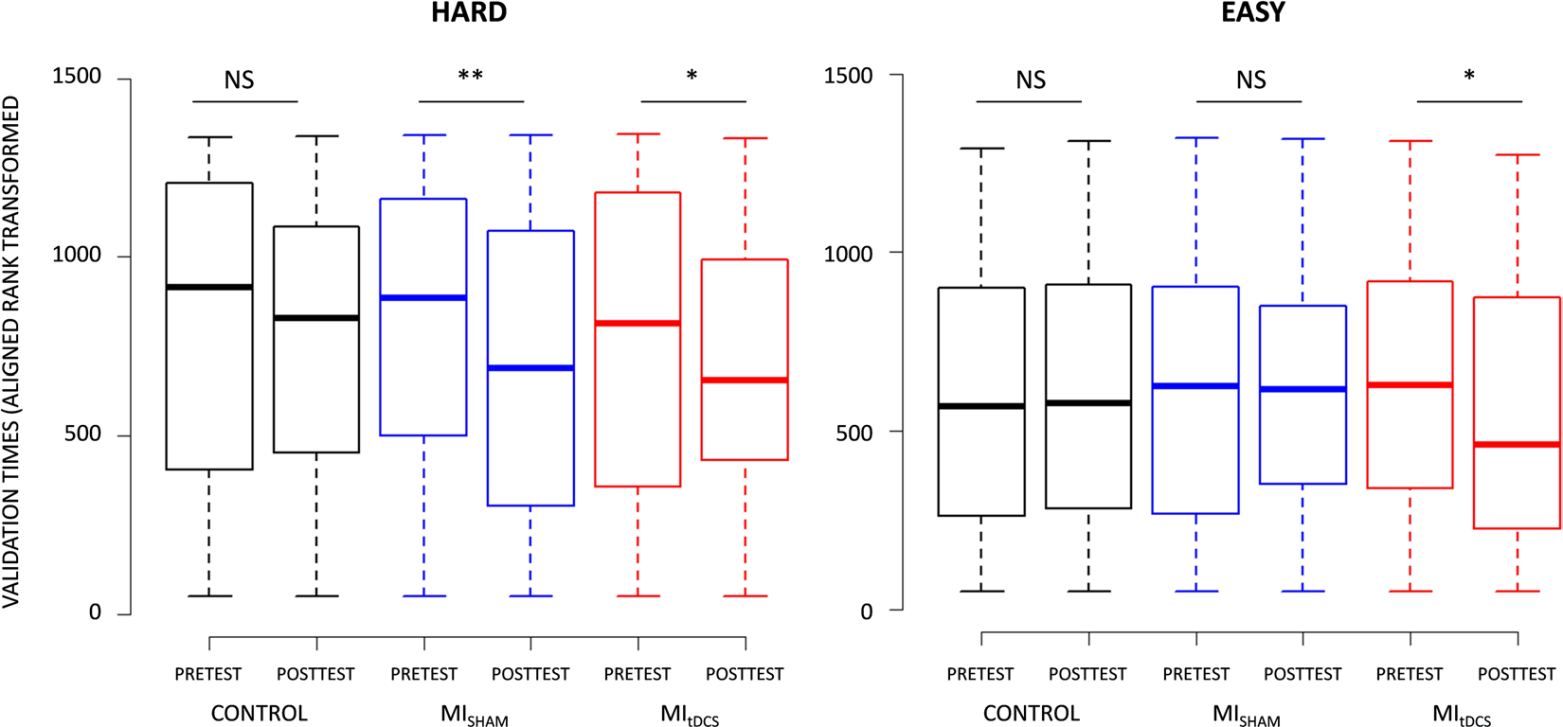
Effect of Control, MI_SHAM_ and MI_tDCS_ conditions on Easy and Hard targets validation.

A CONDITION × TEST × DIFFICULTY interaction was also present (F_(2,1235)_ = 4.09, p_(ART)_ = 0.01, partial η^2^ = 0.07). Validation times of Hard targets decreased from the Pretest to the Posttest under both MI_tDCS_ (Pretest: 4.97 s ± 2.25, Posttest: 4.31 s ± 1.31; p_(ART)_ = 0.03) and MI_sham_ (Pretest: 4.91 s ± 1.66, Posttest: 4.38 s ± 1.38; p_(ART)_ = 0.002), but not under Control (Pretest: 5.06 s ± 1.98, Posttest: 4.99 s ± 1.84; p_(ART)_ > 0.05). Posttest validation times of Hard targets were lower during MI_tDCS_ than during Control (p_(ART)_ = 0.03) while the comparison between MI_sham_ and Control approached the statistical threshold (p_(ART)_ = 0.07) (Fig. 2A). Noteworthy, validation times of Easy targets decreased from the Pretest to the Posttest only after the MI_tDCS_ training (Pretest: 4.04 s ± .79, Posttest: 3.82 s ± .76; p_(ART)_ = 0.02, Fig. 2B). The CONDITION × TEST × DIRECTION interaction approached the statistical threshold (F_(2,1235)_ = 2.50, p = 0.08). Lastly, no CONDITION × TEST × LATERALITY interaction emerged (F_(4,1235)_ = 0.90, p > 0.05).

In contrast to PLAN, DIFFICULTY and LATERALITY (F_(1,1235)_ = 77.92, p < 0.001, partial η^2^ = 0.01; F_(1,1235)_ = 228.41, p < 0.001, partial η^2^ = 0.06; F_(2,1235)_ = 19.34, p < 0.001, partial η^2^ = 0.01, respectively), there was no main effect of DIRECTION on validation times (F_(1,1235)_ = 0.03, p > 0.05). Participants overall exhibited longer validation times for Behind (4.22 s ± 1.88) compared to Ahead (4.04 s ± 1.13) targets (p_(ART)_ = 0.002), and comparison between Behind targets compared to Neutral approached the statistical threshold (Neutral: 4.08 ± 1.14; p_(ART)_ = 0.09). Hard targets also involved longer validation times than Easy targets (Hard: 4.91 s ± 2.30; Easy: 4.05 ± 1.00; p_(ART)_ < 0.001), while both Dominant (4.16 s ± 1.47) and Non-dominant (4.20 s ± 1.63) targets involved longer validation times than Neutral (3.94 s ± 1.03) targets (p_(ART)_ = 0.02 and p_(ART)_ = 0.01, respectively).

**Figure 3:**
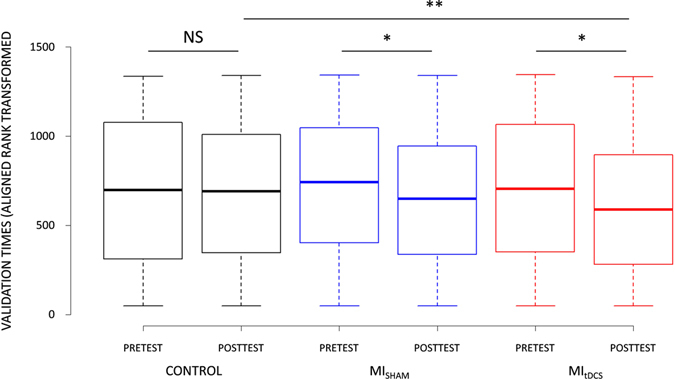
Effect of Control, MI_SHAM_ and MI_tDCS_ conditions on postural task validation.

The factorial analysis yielded a CONDITION × TEST interaction (F_(2,1235)_ = 11.80, p < 0.001). Validation times were reduced during the Posttest compared to the Pretest under both MI_tDCS_ (Pretest: 4.23 s ± 1.47, Posttest: 3.86 s ± .91; p_(ART)_ = 0.002) and MI_sham_ (Pretest: 4.16 s ± 1.26, Posttest: 3.93 s ± 1.08; p_(ART)_ = 0.02), but remained similar during Control (Pretest: 4.15 s ± 1.25, Posttest: 4.06 s ± 1.16; p_(ART)_ > 0.05). Validation times under MI_tDCS_ were lower compared to Control during the Posttest (p_(ART)_ = 0.01), but there was no difference in Posttest validation times between MI_tDCS_ and MI_sham_, nor between MI_tDCS_ and Control (p > 0.05, Fig. 3 ).

## Discussion

This study was designed to investigate the effects of MIP on postural control and determine the additional benefits when paired with anodal tDCS over M_1_. Data revealed *i*) a significant decrease of the time required to perform the postural task after MIP, and *ii*) greater performance gains when MIP was coupled with anodal tDCS when the task involved high demands in terms of postural adjustments.

While there is ample evidence that MIP contributes to improve motor performance and promote motor learning, its potential benefits on the postural control have been far less examined. Our data confirmed that MIP might significantly enhance motor performance of a complex postural task. Previous behavioral studies focusing on the impact of MIP on postural control (usually incorporated in a locomotor task) used longitudinal assessments and involved several training sessions per week, within a span of at least 2 weeks^11,33–37^. Interestingly, the present study extends these findings and provides evidence that a small amount of MIP without any physical practice might be sufficient to impact the postural control. The short-term performance gains (time decrease of 8%) observed after only one single MIP session of 12 min were slightly lower than those reported by Choi *et al*.^7^ after 15 imagery sessions of 30 min (time decrease of 14%) for a postural task requiring to reach and validate targets located forward. Malouin *et al*.^38^ underlined the potential of one single session of MIP to elicit significant enhancement of a functional movement including postural skills in stroke patients, i.e., a greater loading on the affected limb while standing up and sitting down. In this study, the improvement on limb loading was recorded consecutively to 30 min of MIP. As patients also benefited from some physical execution trials in addition to MIP (physical practice/mental practice ratio of 1 to 5), the present results pioneer that a single session of 12 min of MIP without any physical practice may be effective to improve postural control.

It is commonly agreed that the neurofunctional equivalence between motor imagery and actual execution of the same movement is the most plausible explanation for MIP effects on motor performance^19,21^. In a recent study investigating the neural networks involved during motor imagery of a postural task, Taube *et al*.^39^ reported activations in the putamen, the cerebellum, and the supplementary motor area, which play a crucial role during postural control^40,41^. These regions were also found to be strongly involved during the kinesthetic modality of imagery^42,43^. Although this remains a working hypothesis, we assume that 12 min of MIP using kinesthetic imagery would predominantly engage these task-specific networks, and therefore prompt performance gains.

A second finding of this study is that applying anodal tDCS over M_1_ during MIP resulted in slightly greater performance compared to both MIP with sham tDCS and Control conditions. The additional effect of anodal tDCS on MIP-induced performance improvements confirms the results by Foerster *et al*.^29^ where a single session of MIP yielded to significant performance gains only when imagery was coupled with tDCS. Using a finger sequential task, Saimpont *et al*.^30^ further showed that MIP contributed to improve performance, however with results when tDCS was simultaneously applied over M_1_. Yet, only these two experimental studies focusing on upper-limb motor performance investigated the combined effect of both tDCS and MIP. The present study tends to support the effectiveness of applying tDCS over M_1_ during MIP to improve postural control. How anodal tDCS could increase the impact of MIP may be explained by greater stimulation of M_1_. As enhanced cortical excitability is known to facilitate motor learning^25^, combining both techniques is likely to improve the strengthening of synaptic transmission. Specifically, Nitsche *et al*.^44^ showed that applying tDCS over M_1_ might modulate the motor cortical transmitter system by reducing the activity of the gamma-aminobutyric acid (GABA)-ergic system. Whether a similar mechanism is effective when combining MIP with anodal tDCS yet remains a working hypothesis awaiting experimental investigation. Given the diffuse nature of tDCS^45^, however, some areas adjacent to M_1_ may also have been stimulated concomitantly. In particular, we cannot totally rule out that the SMA, which plays an important role in postural control^40,41^, and is activated during motor imagery of postural tasks^39^, was also stimulated. Although Antal *et al*.^46^ reported a decreased activation in this region after anodal tDCS over M_1_, divergent findings have been found when directly applying tDCS over the SMA. While Kaminski *et al*.^47^ showed that stimulating the SMA impaired motor learning of a dynamic balance task, some recent data provided evidence of substantial performance gains in different motor tasks^48–51^. Taken together, the contribution of the SMA, although being debated, can thus not be completely ruled out in the present study.

The beneficial effects of applying tDCS over M_1_ during MIP were mainly observed when looking at the fine postural adjustments required by the task. The present paradigm involved targets located at different directions with two levels of difficulty (Easy and Hard targets), both forward and backward (Ahead and Behind targets). Performance improvements during both MI_tDCS_ and MI_sham_ conditions were recorded for Hard targets, whereas the time needed to complete Easy targets was reduced only under the MI_tDCS_ condition. Different strategies might be used to maintain balance. Validating Hard targets may imply a shift of the CoP soliciting the hip joint, whereas minor deviations, such as those required to validate the Easy targets, might primarily manage through ankle involvement^52^. Furthermore, one can postulate that the margin for performance improvements was higher for Hard than for Easy. Data therefore demonstrate that only MI_tDCS_ resulted in performance gains for both types of targets. Likewise, both conditions yielded to significant performance enhancement for Ahead targets, but only MI_tDCS_ improved the postural control for trials involving Behind targets. Considering the importance of rehearsal in motor learning, backward CoP displacement are more complicated than those in the forward direction where daily activities are more frequently oriented (e.g., locomotion, prehension). Furthermore, while movements in the forward direction might benefit from a broad support area (i.e., the 5 toes and the midfoot), backward movements rely on a narrow base (i.e., the heel), hence increasing the difficulty of validating Behind targets^53^. Taken together, these results suggest that the cortical activity in M_1_ induced by tDCS during MIP might contribute to reinforce the positive effect of MIP and result in greater performance for particularly demanding postural adjustments. As suggested by Foerster *et al*.^29^ and Saimpont *et al*.^30^, tDCS may therefore appear a promising stimulation method to potentiate the effects of MIP.

As with all experimental research, the present pilot study has some limitations which should be overcome in future experiments. As mentioned previously, applying tDCS over M_1_ might have activated other cortical adjacent regions, such as the SMA. One alternative to resolve this issue might be to stimulate M_1_ and the SMA in two separate sessions, as shown by Foerster *et al*.^29^. From a pure methodological viewpoint, however, Lang *et al*.^54^ argued that tDCS stimulation is likely to induce widespread bi-directional changes in regional neuronal activity, hence resulting in profound effects on the overall level of regional activity in many other brain regions. Future studies should certainly combine electroencephalographic measurements to tDCS stimulation in order to better understand and improve the neurophysiological correlates of the brain activity during each experimental condition. Within a behavioral perspective, additional postural variables (e.g., sway length) could have been informative regarding the qualitative aspects of the experimentally-induced changes in postural control. Here, the data revealed that both MI_tDCS_ and MI_sham_ led to performance gains, as shown by decreased time to validate targets. Interestingly, Choi *et al*.^7^ reported that similar effects were not paralleled by reduced sway length, hence suggesting that participants took less time to perform the task, but were not necessarily more accurate. Replicating the study while recording postural sway might thus be relevant to clarify this issue. Third, future studies might include retention tests in order to see whether the effects observed after one single session of MIP are strictly maintained over time. Fourth, the experimental design did not involve a tDCS “only” condition. Based on recent findings by Saimpont *et al*.^30^, however, coupling MIP with tDCS applied over M_1_ resulted in significant greater performance compared to tDCS alone. Finally, other experimental conditions should ideally be included in future experimental designs to adequately frame and conclude about the relevance of combining MIP and tDCS. A pure physical practice condition might be necessary to compare the effects of physical and mental practice. Investigating the effects of tDCS during different ratios of physical and MIP (see Reiser *et al*.^55^) might also be very informative to improve the reasoning about the functional relevance of combining MIP with tDCS.

Overall, our results provide evidence of short-term beneficial effects of both MIP combined with tDCS and MIP alone on postural control. Combining MIP with tDCS applied over M_1_ might further appear a promising approach to enhance fine postural skills. Determining whether combining tDCS during MIP leads to greater performance than performing MIP before/after a tDCS session may be an exciting focus of research for future studies. More generally, these results have theoretical and practical applications in both motor learning and (neuro)rehabilitation. Clinical studies should notably address the relevance of combining both methods as an adjunctive rehabilitation practice to classical physical therapy for regaining postural skills in easily fatigable persons (e.g., elderly people) and neurologic populations (e.g., stroke patients).

## Methods

### Participants

Fourteen right-handed healthy participants (8 males, 6 women, mean age 25.78 ± 3.76 years) voluntarily participated in this double-blind study, which was approved by the Research Ethics Committee of the Center of Research and Innovation in Sport (University Claude Bernard Lyon 1). Handedness was surveyed by a brief self-report patient-based questionnaire adapted from Olfield^56^. Only persons reporting a strong dominance of the right hand to perform everyday activities were recruited in the experimental design. Contra-indication to tDCS and musculoskeletal disorders were considered exclusion criteria. Shoe size above 10.5 (US size) was also an exclusion criterion to avoid feet extending beyond the postural tool assessment area. The experiment was performed in accordance with relevant guidelines and regulations. Informed consent in agreement with the terms of the Declaration of Helsinki (2013) was obtained from all participants. The procedure of the experiment and the tasks were individually explained, but no information was provided about the objectives of the study or the variables of interest before experimentation began. Participants were debriefed at the end of the study. Due to the novelty of the postural task investigated in this pilot study, and the lack of corresponding information in the literature, we were not able to run a power analysis to determine sample size *a priori*.

### Design

The experiment took place in a quiet room, lit by homogeneous white light, i.e., in stable and reproducible environmental conditions. Two experimental (MI_tDCS_, MI_sham_) and one control (Control) conditions were scheduled, within a test-retest procedure. All sessions were randomized and separated by one week. Participants were thus randomly assigned to one of the 6 possible session orders, to avoid both practice and order effects. Before engaging in the experimental design, participants were subjected to a pre-visit to assess their MI vividness (i.e., the clarity of the mental images and the intensity of the sensations perceived during MI) through the Kinesthetic and Visual Imagery Questionnaire (KVIQ-10)^57^. The questionnaire includes 10 items corresponding to 5 basic movements that participants must perform and then mentally imagine using both visual and kinesthetic imagery. For each movement, participants were requested to rate the clarity of the images (visual imagery) or the intensity of the sensations they perceived (kinesthetic imagery) during the imagined movement using, a 5-point analog scale. A score of 5 corresponded to the highest level of MI (images as clear as seeing and sensations as intense as perceived when performing the action). Conversely, a score of 1 corresponded to the poorest imagery (no images/no sensations).

**Figure 4:**
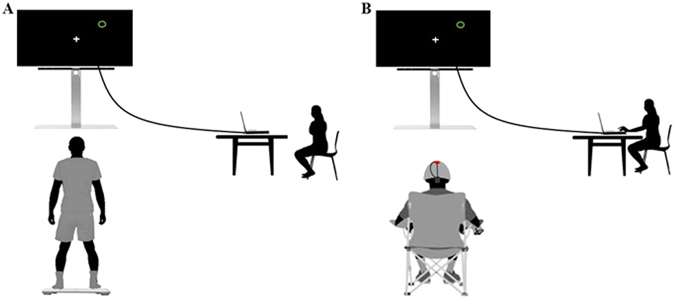
Actual performance and MI of the postural task. (**A**) Actual performance assessed during Pre and Post-Test. (**B**) MI of the task during MI_tDCS_, MI_sham_ and Control conditions.

**Figure 5:**
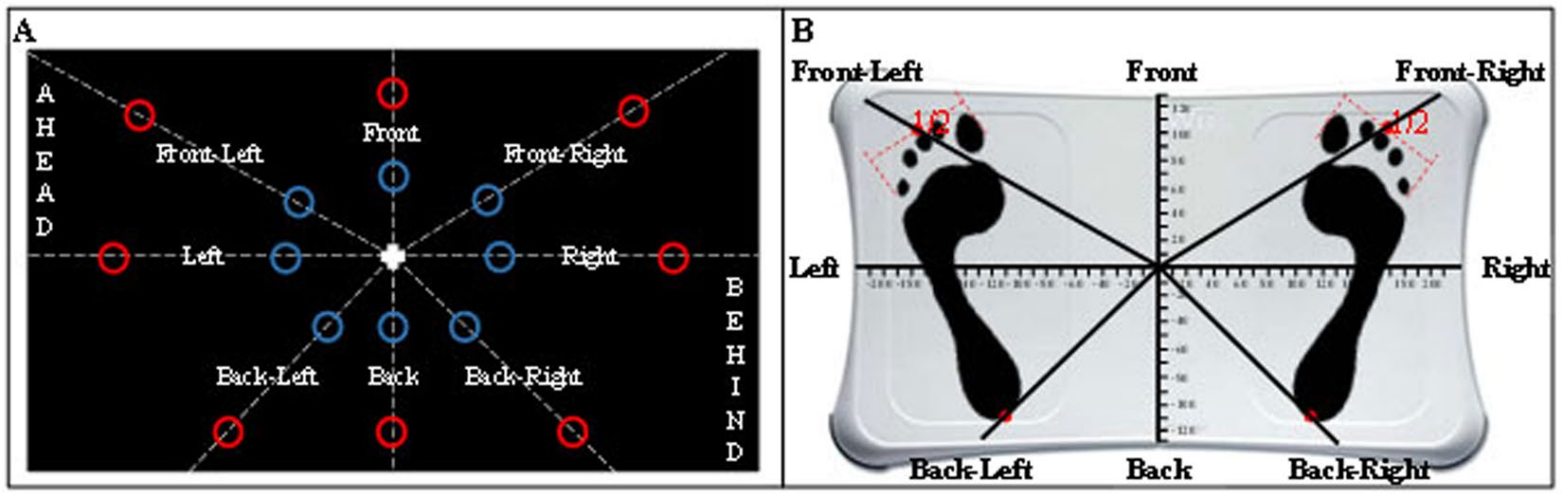
Axes and targets. (**A**) Location of the 16 targets on each of the 8 locations, for easy (blue targets) and hard (red targets) difficulty levels. Note that targets appeared only in green during the experimentation. (**B**) Measurement of individual support polygon for personalized theoretical stability limitations and directions definition.

For both experimental and Control conditions, participants were subjected to identical pre- and post-tests, and consisted on a complex postural task (Fig. 4A). Participants had to shift their center of pressure (CoP), without lifting their feet nor losing balance, to validate 16 targets that appeared in 8 different directions. CoP was measured using a Wii Balance Board (Nintendo, USA), which has shown consistent concurrent validity when tested with healthy and young people^58^. When compared to a laboratory force plate, Clark *et al*.^58^ found good test-retest and between-device intra-class correlation coefficient (>0.66). Moreover, the use of the Wii Balance Board as a posture measure tool was validated in young people^31,32^, elderly^59^, persons with Parkinson's disease^60^, visual impairment^61^, and reconstructed anterior cruciate ligament^62^. A software (Polyenco, France) was specifically developed to connect the Wii Balance Board to the computer in order to display the CoP position in a form of a cross on the screen, and randomly picture the targets to validate (Fig. 4A). Targets were located in 8 directions (Front, Back, Left, Right, Front-Left, Front-Right, Back-Left and Back-Right), at two levels of difficulty (easy and hard) directly based on the individual sustentation polygon (Fig. 5A). Coordinates of feet position were measured at six different points (thumbs, pinky fingers and heels) to delimitate the individual theoretical stability limitations in both axes, and to fix the places where targets randomly appeared for each participant. To respect the individual characteristics of the natural stance position, participants were asked to stand comfortably and motionless during measurement. Coordinates were recorded once, and then reused for each condition. Back-Left and Back-Right axes were the lines connecting the center to the heels. Front-Left and Front-Right axes connected the center to the point of equilibrium between toes (i.e., half distance between the big toe and the pinky toe, Fig. 5B). Easy targets corresponded to 20% of the theoretical maximum stability limitation, regardless of the forward/backward direction, while hard targets were placed at 50% of this limit. All Ahead targets located in the forward direction, as well as all Behind targets, located in the backward direction, were therefore either considered easy or hard. The 16 targets (one per location and difficulty) had to be validated by placing the CoP into each target during 3 s (time was cumulated if participants moved outside the target area). Participants should systematically move back to the center before validating the next target, and were never allowed to lift their feet throughout the testing session.

Immediately after the Pretest, participants were subjected either to a 12 min experimental (MI_tDCS_ or MI_sham_) or Control condition. During each experimental condition, participants were requested to mentally imagine the postural task while receiving anodal tDCS (MI_tDCS_ condition) or sham tDCS (MI_sham_ condition) over M_1_. To avoid muscular contractions and fatigue due while remaining motionless in a standing position for a long time, the MI session was performed while participants seated on a chair, at the exact place where they actually performed the postural task on the Wii Balance Board. They were asked to imagine that they validated each target by mentally shifting their body weight, through kinesthetic imagery, as well as to focus on tactile sensation of their feet on the Wii board. Using a custom made program, individual targets, similar to those presented during both pre- and post-test, randomly appeared on the screen. Participants verbally indicated when the target was validated and the investigator clicked to make the next target appear (Fig. 4B). As during both the pre- and post-test, they were asked to imagine moving back to the center before mentally validating the next appearing target. After both experimental conditions, participants rated on a 5-point Likert scale (graduated from 1: no sensation, to 5: as intense as during actual performance) the intensity of the sensations perceived during the 12 min of MIP. In the Control condition, participants played a manual precision interactive game while receiving sham tDCS.

### Apparatus

tDCS was delivered with a constant current stimulator (STARSTIM, Neuroelectrics) using two sponge electrodes impregnated with a saline-soaked solution. To increase cortical excitability of lower-limbs, the anode electrode was placed medially over the leg areas of both M_1_^63^, which are located laterally to the vertex^64,65^. To cover both left and right hemispheres, the anode (25 cm², current density 0.04 mA/cm²) was placed at Cz^66^, with reference to the International 10/20 system. The cathode electrode (35 cm², current density 0.028 mA/cm²) was placed on the middle of the forehead. To avoid discomfort, current ramped up during 40 s until it reached 1 mA, and then ramped-down during 40 s after 12 min of stimulation. For blinding effect, the current was ramped up to 1 mA and down to 0 during the first and last 40 s of the sham conditions, but was nil during the remaining time. At the end of each session, participants were asked whether they received a real stimulation and could answer “Yes”, “No”, or “I don’t know”. We performed conformity Chi-squared test on the proportions of participants’ “Yes” and “No” answers for each session against proportions corresponding to the chance level (50%). Participants who responded “I don’t know” were not included in this analysis since they acknowledged not being able to discriminate sham from real stimulations. The conformity Chi-squared test were not statistically significant (all p > 0.05).

### Data analysis

The time elapsed from target display to target validation (i.e., validation time) was the dependent variable quantifying motor performance. In addition to CONDITION (MI_tDCS_, MI_sham_, and Control) and TEST (Pretest; Posttest), we included DIFFICULTY (Easy: easy targets; Hard: hard targets), LATERALITY (Dominant: front-right, right and back-right targets, Non-Dominant: front-left, left and back-left targets; Neutral: front and back targets), PLAN (Ahead: front-left, front and front-right targets; Behind: back-left, back and back-right targets; Neutral: left and right targets), and DIRECTION (Antero-posterior: front and back targets; Medio-lateral: left and right targets; Mixed: front-left, front-right, back-left and back-right targets) as independent variables (see Fig. 2A).

We used R^67^ and the packages *lme4* ^68^ and *ARTool* ^69^ to perform a nonparametric factorial analysis of motor performance data. Due to deviations from normality (Q-Q plots), we implemented a validated aligned rank transform (ART) procedure^70^. The ART procedure consists in a preliminary step of data alignment based on the mean estimates of main and interaction effects of a given factorial model, followed by rank assignment (for further details, see Wobbrock *et al*.^70^). We applied the ART to a mixed linear model with validation times as response variable. As fixed effects, we entered the independent variables (with interaction term). As random effect, we entered the by-subject random intercept. As post-hoc investigations, we used contrast tests with ART (least square means difference) and ran a systematic investigation of the three-way interactions involving CONDITION, TEST and one of the target location factor (i.e., DIFFICULTY, DIRECTION, LATERALITY and PLAN). The main effect of DIFFICULTY, DIRECTION, LATERALITY and PLAN was also investigated. The statistical significance threshold was set for a type 1 error rate of 5%. Holm's corrections for multiple comparisons were applied to control the false discovery rate^71^. Effect sizes were reported for each significant result. Small effect sizes were, however, expected due to the small sample size, the short duration of the experimental design (investigating immediate effects on performance at the session level), as well as the variable of interest (i.e., validation of the targets with limited magnitude of performance gains) and the three-way interactions.
